# Supplementary material for: In vitro experimental conditions and tools can influence the safety and biocompatibility results of antimicrobial electrospun biomaterials for wound healing
Source: PLoS One. 2024 Jul 1;19(7):e0305137. doi: 10.1371/journal.pone.0305137 (PMC11216574; doi:10.1371/journal.pone.0305137)
Supplement: S1 File — (PDF) [file pone.0305137.s001.pdf]

RTCA measurement for indirect extract exposure assay

The RTCA experiment was performed as described in the Methods section (Indirect extract exposure assay) using PF cells with a higher passage number (p12). The overall graph shape was similar, but the cell index value was lower when using cells with higher passage numbers (S1 Fig).

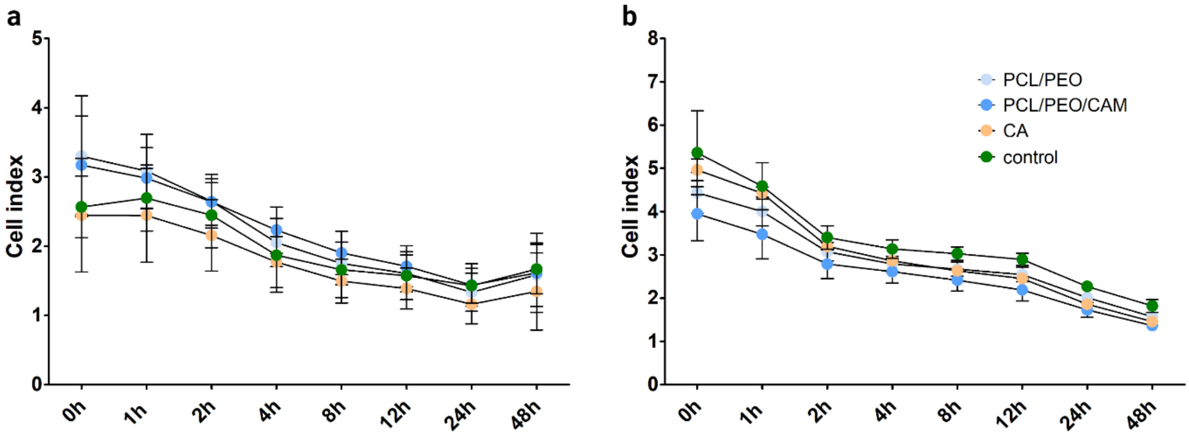

S1 Fig. Results of RTCA indirect ES fiber mat extract exposure assay performed on PF cells at passage numbers 12 (a) and 5 (b). Key: CA - Cellulose acetate filters; Control – untreated cells growing on the bottom of the well plate; PCL/PEO - electrospun (ES) fiber made from polycaprolactone and polyethylene oxide; PCL/PEO/CAM - ES fibes made from polycaprolactone and polyethylene oxide containing chloramphenicol. Error bars represent the mean of three biological replicates.
