# Supplementary material for: In vitro experimental conditions and tools can influence the safety and biocompatibility results of antimicrobial electrospun biomaterials for wound healing
Source: PLoS One. 2024 Jul 1;19(7):e0305137. doi: 10.1371/journal.pone.0305137 (PMC11216574; doi:10.1371/journal.pone.0305137)
Supplement: S2 File — (PDF) [file pone.0305137.s002.pdf]

Investigation of possible adsorption and/or absorption of the MTS reagent to ES fibers

To exclude the possibility of misinterpretation of the results due to the possible nature of the ES fiber material to adsorb and/or absorb the MTS reagent, two experiments were designed. For the first experiment, BHK-21 cells were seeded in a 24-wellplate at a density of 5×10^4 cells/well in 1.25 mL medium. The seeded cells were then incubated at 37°C in a 5% CO_2 atmosphere for 24 h. Then, pieces of ES fibers and CA filter in the size of 1.5 x 1.5 cm were placed into wells with cells and 125 μL of MTS Cell Proliferation reagent was then added to each well with cells and ES fiber material. An additional 1 h of incubation at 37°C in a 5% CO_2 atmosphere was performed until colour change was observed. Samples (200 μL) from a 24-wellplate were transferred to a 96-wellplate, resulting in technical duplicates. Absorbance was measured using a microplate reader at an optical density of 490 nm. No significant differences were observed between the groups (S2 Fig).

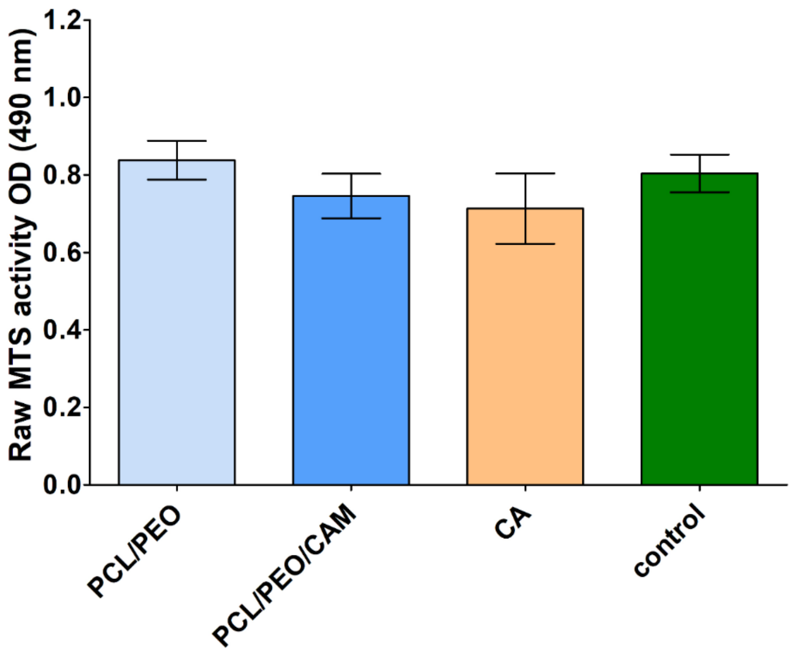

S2 Fig. Results of possible MTS reagent binding to the ES fibers in the presence of BHK-21 cells. Key: CA - Cellulose acetate filters; Control – untreated cell growing on the bottom of the well plate; PCL/PEO - ES fiber made from polycaprolactone and polyethylene oxide; PCL/PEO/CAM - ES fibers made from polycaprolactone and polyethylene oxide containing chloramphenicol. Error bars represent the mean of three biological replicates.
