# Supplementary material for: In vitro experimental conditions and tools can influence the safety and biocompatibility results of antimicrobial electrospun biomaterials for wound healing
Source: PLoS One. 2024 Jul 1;19(7):e0305137. doi: 10.1371/journal.pone.0305137 (PMC11216574; doi:10.1371/journal.pone.0305137)
Supplement: S3 File — (PDF) [file pone.0305137.s003.pdf]

Investigation of possible adsorption and/or absorption of the MTS reagent to ES fibers

The leftover medium with ES fibers and CA filter from the S2 Fig experiment transferred to a clean 24-wellplate to remove the cells. An additional 1 h of incubation was performed. Before transferring the ES fibers to a clean wellplate with old medium, the fibers were photographed to capture the visual colouration of the fibers possibly caused by the binding of the MTS reagent. The photography step was repeated after the second incubation without cells. Next, the ES fibers were washed with 1xPBS to remove the possible redness caused by phenol red from the medium and the fibers were photographed again. No drastic colour change was observed, indicating that the MTS reagent may only slightly adsorb and/or absorb the tested ES fibers (S3 Fig).

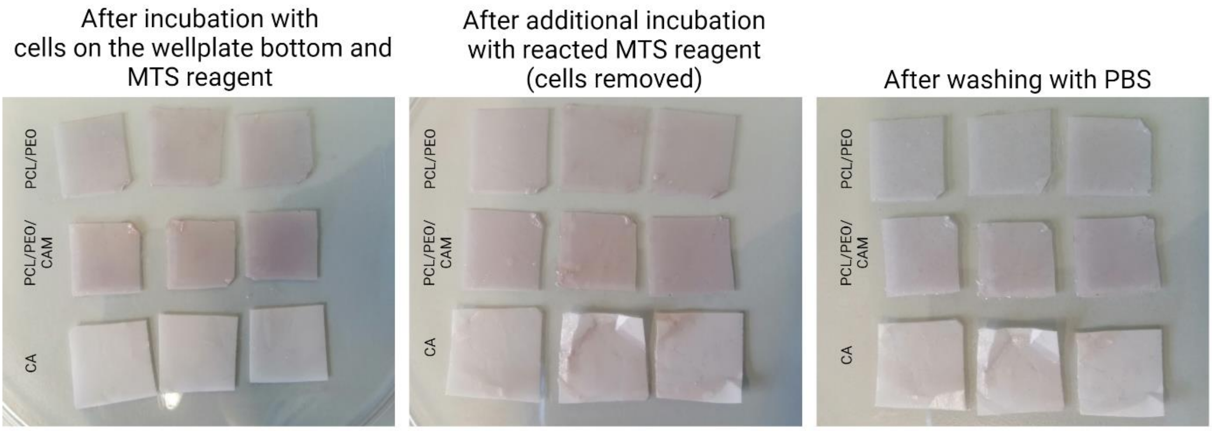

S3 Fig. Photographs of the tested ES fibers taken after each experimental step to visually detect the possible adsorption and/or absorption of the MTS reagent.
