# Supplementary material for: In vitro experimental conditions and tools can influence the safety and biocompatibility results of antimicrobial electrospun biomaterials for wound healing
Source: PLoS One. 2024 Jul 1;19(7):e0305137. doi: 10.1371/journal.pone.0305137 (PMC11216574; doi:10.1371/journal.pone.0305137)
Supplement: S4 File — (PDF) [file pone.0305137.s004.pdf]

MTS measurement of remaining cells on the bottom of wellplate

The experiment was performed as described in the Methods section (the Direct contact method). After removing the cell culture inserts, the ES fiber material was transferred to a new 24-wellplate and 500 μL DMEM medium was added. In the control wells with cells, the old medium was replaced with fresh medium. Wells from where inserts were removed, the old medium was changed to 500 μL of fresh medium and 50 μL MTS Cell Proliferation reagent was then added to each well with cells and ES fiber material. An additional 1 h of incubation was performed until colour change was observed. Samples (200 μL) from a 24-wellplate were transferred to a 96-wellplate, resulting in technical duplicates. The absorbance was measured using a microplate reader at an optical density of 490 nm.

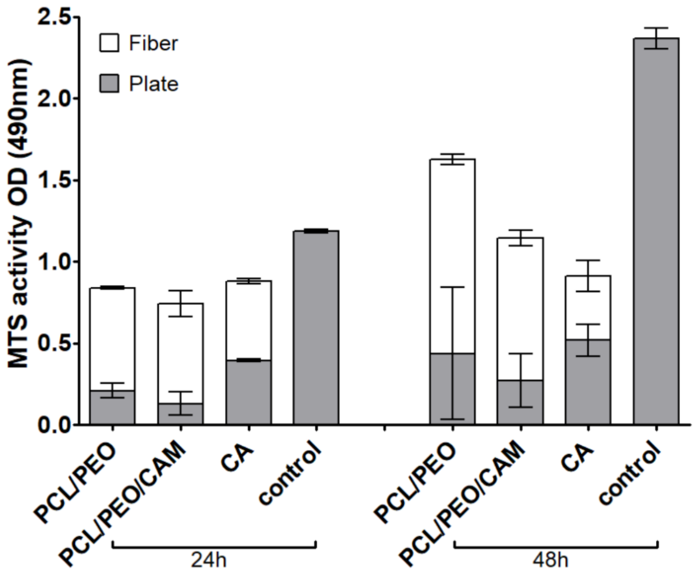

S4 Fig. MTS activity was measured from the ES fibers and the well plate bottom after the removal of the cell culture inserts with ES fibers at 24 h and 48 h. Key: CA - Cellulose acetate filters; Control – untreated cell growing on the bottom of the well plate; PCL/PEO - ES fiber made from polycaprolactone and polyethylene oxide; PCL/PEO/CAM - ES fiber made from

polycaprolactone and polyethylene oxide containing chloramphenicol; OD- optical density. Error bars represent the mean of three biological replicates.
