# Supplementary material for: In vitro experimental conditions and tools can influence the safety and biocompatibility results of antimicrobial electrospun biomaterials for wound healing
Source: PLoS One. 2024 Jul 1;19(7):e0305137. doi: 10.1371/journal.pone.0305137 (PMC11216574; doi:10.1371/journal.pone.0305137)
Supplement: S5 File — (PDF) [file pone.0305137.s005.pdf]

Light microscopy imaging

The experiment was performed as described in the Methods section (the Direct contact method). After removing the cell culture inserts with ES fibers, the wells were imaged using a Zeiss Primovert inverted light microscope.

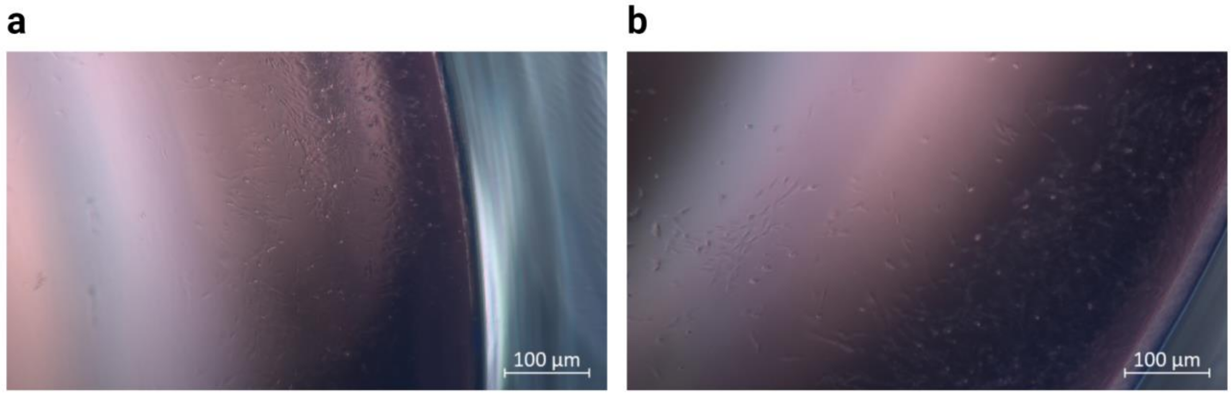

S5 Fig. Baby hamster kidney (BHK-21) cells growing on the bottom of the plate after removal of the cell culture inserts with PCL/PEO (a) and PCL/PEO/CAM (b) ES fibers after 48 h of incubation.
