# Supplementary figures and images for: In vitro experimental conditions and tools can influence the safety and biocompatibility results of antimicrobial electrospun biomaterials for wound healing
Source: PLoS One. 2024 Jul 1;19(7):e0305137. doi: 10.1371/journal.pone.0305137 (PMC11216574; doi:10.1371/journal.pone.0305137)

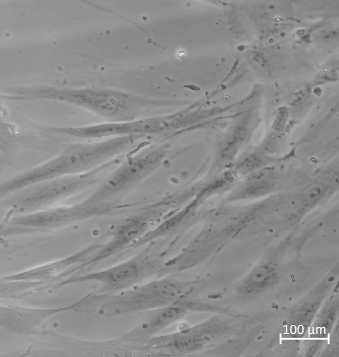

Supplement: S8 File — Raw SEM and light microscopy images. (ZIP) [file pone.0305137.s008.zip › SEM and light microscopy images/Fig 1 PF.png]

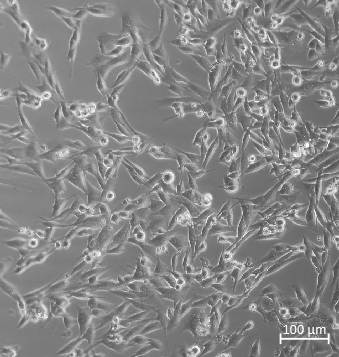

Supplement: S8 File — Raw SEM and light microscopy images. (ZIP) [file pone.0305137.s008.zip › SEM and light microscopy images/Fig1 BHK.png]
