# Supplementary material for: In vitro experimental conditions and tools can influence the safety and biocompatibility results of antimicrobial electrospun biomaterials for wound healing
Source: PLoS One. 2024 Jul 1;19(7):e0305137. doi: 10.1371/journal.pone.0305137 (PMC11216574; doi:10.1371/journal.pone.0305137)
Supplement: Attachment — Submitted filename: Response Letter290424.docx [file pone.0305137.s010.docx]

**Response Letter to reviewers**

Ref: PONE-D-24-00754

Academic Editor

Isha Mutreja
*PlosOne*

Prof Karin Kogermann

Institute of Pharmacy, Faculty of Medicine

University of Tartu, Nooruse 1, 50411 Tartu, Estonia

E-mail: [karin.kogermann@ut.ee](mailto:karin.kogermann@ut.ee)

Dear Prof Dr Isha Mutreja,

We would like to thank the Reviewers for their thoughtful comments on our paper entitled „***In vitro* experimental conditions and tools can influence the safety and biocompatibility results of antimicrobial electrospun biomaterials for wound healing**”. Our answers and corresponding corrections to the manuscript based on their comments are written in a point-by-point response letter to the Reviewers. The Reviewer’s comments (**bold letters**) and our responses (in regular letters) are given below. We have also uploaded a revised manuscript and highlighted the places where the modifications have been performed using yellow highlighting. We have also followed the Journal´s instructions for formatting the manuscript (these formatting changes are not highlighted) and made all raw data available in Supplementary files.  We have also uploaded revised **Figures 3, 6, 7** and **Supplementary Materials**.

We hope that the revised version of our manuscript meets the criteria to be published in the *PlosOne.*

On behalf of all authors,

Yours sincerely,

Karin Kogermann

Professor of Physical Pharmacy

Head of Institute of Pharmacy,

University of Tartu, Faculty of Medicine,

Institute of Pharmacy,

Nooruse 1, 50411 Tartu, Estonia

Tel. +372-56509455

Email [karin.kogermann@ut.ee](mailto:karin.kogermann@ut.ee)

Website: [kogermannlab.com](https://kogermannlab.com/)

29.04.2024

**Reviewer 1**:

**This is a review of PONE-D-24-00754, a manuscript comparing in vitro cytotoxicity testing methods of electrospun polycaprolactone and polyethylene oxide membranes with chloramphenicol. This is a very well described, technically sound article describing different cytotoxicity testing methods. This manuscript is useful for those performing similar cytotoxicity testing as it describes a clear framework for thinking through these important in vitro experiments. I support the publication of this paper after the following comments are addressed.

Abstract

Q1: I would edit the sentence containing “true biocompatibility” to mention in vitro as one could argue that “true biocompatibility” requires animal testing.**

R1: We have modified the abstract of the manuscript and the word “true” was removed from the sentence to make it clearer. The text now reads:

Line 25: “We found that while extract exposure assays are suitable for the initial testing, the biocompatibility of the biomaterial is revealed in *in vitro* direct contact assays where cell interactions with the ES wound dressing are evaluated.”

**Intro**

**Q2: This is only a suggestion, but the first paragraph could be shortened and collapsed into the second paragraph. It is likely that the readers of this manuscript have relevant background in biomaterials to not need the definition of biomaterials or drug delivery systems.**

R2: Thank you for your suggestion. However, after careful consideration and reading reflections from both Reviewers, we decided to leave the first paragraph on purpose longer to give readers who may not have such good background knowledge a chance to understand the topic without looking for additional external information. Indeed, for the experts in the field it is a short summary about the topic they are already familiar.

**Q3: In the fourth paragraph, I would mention the purpose of the microscopy-based methods (LIVE/DEAD, etc) to give better context.**

R3: In our study, microscopy was used to study cell morphology when growing in contact with ES fiber mats. The morphological changes give excellent picture about the physiological response of the cells, that is why we used confocal microscopy. In addition, when used in combination with suitable staining methods (e.g. live/dead staining) proof about cell viability, can be collected. We modified two sentences (and added relevant references), one in the fourth paragraph and the other at the end of the fifth paragraph. Sentences read now as follows:

Line 79: “After an exhaustive literature search, we found that both extract exposure and direct contact methods have been widely used, but not always together with microscopy, which would give better understanding about cell morphological changes when in contact with material. It has been shown that fluorescent staining together with microscopy enables to enlighten the structure of cells and depending on the selected fluorophore and its sensitivity and selectivity^14^.”

Line 103: “The results of the cytotoxicity assay were complemented by confocal microscopy together with DAPI and Alexa 568 staining in order to illustrate the cell morphology.”

Reference:

Alhede, M., Stavnsbjerg, C. & Bjarnsholt, T. The use of fluorescent staining techniques for microscopic investigation of polymorphonuclear leukocytes and bacteria. *APMIS* **126**, 779–794 (2018). DOI: [10.1111/apm.12888](https://doi.org/10.1111/apm.12888)

**Methods**

**Q4: Information should be added about how the samples were or were not sanitized/sterilized.**

**Q5: Any washing of the samples after preparation, considering the solvents used, should be mentioned.**

R4 and R5: The samples were not additionally sanitized or washed after the preparations to remove any leftover solvents. However, the samples were kept in desiccator above silica gel under vacuum (0%RH) for at least 24 h prior further analyses. Additional sentences about this have been added into the Methods section:

Line 491: “After electrospinning, the fiber mats were kept above silica gel (0% relative humidity) under vacuum in desiccator for at least 24 h prior further analyses. Fiber mats were not additionally washed to dispose any leftover solvents from ES process and were also not additionally sanitized prior cell studies.“

**Q6: The authors should mention why these two particular cells were selected.**

R6: These two sentences were added to the revised manuscript:

Line 468: “The BHK-21 cell line was chosen because it has been used previously by our research group to test ES materials safety^9,57^. The PF cell line was used to add clinical relevance to the study.”

References:

Lanno, G.-M. *et al.* Antibacterial Porous Electrospun Fibers as Skin Scaffolds for Wound Healing Applications. *ACS Omega* **5**, 30011–30022 (2020). DOI: [10.1021/acsomega.0c04402](https://doi.org/10.1021/acsomega.0c04402)

Zupančič, Š. *et al.* Impact of PCL nanofiber mat structural properties on hydrophilic drug release and antibacterial activity on periodontal pathogens. *European Journal of Pharmaceutical Sciences* **122**, 347–358 (2018). DOI: [10.1016/j.ejps.2018.07.024](https://doi.org/10.1016/j.ejps.2018.07.024)

**Results and Discussion**	**Q7: The figures are well-constructed and clear, congrats to the authors. However, Figures 6 and 7 should say “conjugated” – typo.**

R7: Thank you for noticing. We have revised this and uploaded modified Figures 6 and 7.

**Q8: This is only a suggestion, but the authors could add information to their discussion about destructive vs. non-destructive testing methods as it relates to cytotoxicity testing.**

R8: Thank you for your suggestion. We have added a small paragraph about this aspect to the Discussion section. The text reads as follows:

Line 370: “One aspect to consider is whether the method is destructive (endpoint only) or non-destructive (continuous measurement). Endpoint only measurement leaves an information gap between the two time points and is measured from different samples, but continuous monitoring gives much better understanding of cell behaviour over time. Therefore, we used continuous cell monitoring via RTCA in indirect studies to complement the endpoint results of the MTS assay.”

**Q9: The author should mention the degradation kinetics of these membranes as it relates to leaching monomers, etc. and differences herein between direct vs. indirect.**

R9: From our previous studies we know that CAM is quickly released from the PCL/PEO/CAM ES fibers and PEO dissolves in aqueous solutions (Preem et al. 2017 and 2019), but PCL is very durable and remains the same for longer time-period. We have added explanations about this to the revised manuscript and linked it with the choice of the indirect extract exposure method. The text reads now as follows:

Line 365: “From our previous studies, we know that CAM is released fast from the PCL/PEO/CAM ES fibers and much of the PEO is dissolved, but PCL remains the same in aqueous solution^18,21^. Therefore, it is important to use the extract exposure method to understand the toxic effects of the released substances on eukaryotic cells. We showed that the substances released from the PCL/PEO and PCL/PEO/CAM ES fiber mats during the specified time period were safe for the cells (**Figs. 2** and **3**).”

References:

Preem, L. *et al.* Interactions between Chloramphenicol, Carrier Polymers, and Bacteria-Implications for Designing Electrospun Drug Delivery Systems Countering Wound Infection. *Molecular Pharmaceutics* **14**, 4417–4430 (2017). DOI: [10.1021/acs.molpharmaceut.7b00524](https://doi.org/10.1021/acs.molpharmaceut.7b00524)

Preem *et al.* Monitoring of Antimicrobial Drug Chloramphenicol Release from Electrospun Nano- and Microfiber Mats using UV Imaging and Bacterial Bioreporters. *Pharmaceutics* **11**, 487 (2019). DOI: [10.3390/pharmaceutics11090487](https://doi.org/10.3390/pharmaceutics11090487)

**Q10: Another topic, only a suggestion, is discussion of differences between MTS, MTT, resazurin, etc.**

R10: Thank you for the suggestion. After reflecting your comment, we decided not to deeply discuss the differences between MTS, MTT and resazurin, since this was not the main focus of this manuscript and we have referenced relevant papers discussing about these methods in our paper.

**Conclusion
Satisfactory, thank you authors.**

**Reviewer 2:**

**The manuscript titled “in vitro experimental conditions and tools can influence the safety and biocompatibility results of antimicrobial electrospun biomaterials for wound healing” is well written and highlights the necessity of investigating biocompatibility using different cell types. This is important not only for electrospun fibers but also for other nanomaterials and biomaterials for biological and biomedical applications. The manuscript does need major revision prior to acceptance.

1. The title says “antimicrobial electrospun biomaterials” while discussing the cytotoxicity of PCL/PEO electrospun fiber pristine and loaded with Chloramphenicol for wound healing applications but no antimicrobial or wound healing assessments were performed.**

The aim of this study was to develop suitable methods for the *in vitro* safety and biocompatibility testing and highlight the aspects that may affect the results, their interpretation and what should be considered when testing such novel biomaterials. The ES fiber mats containing chloramphenicol that were used as model dressings for our safety studies have already been characterised for their antibacterial activity using different pathogenic bacteria and antimicrobial assays in our previous works (Preem et al. 2017, Preem et al. 2019, Lorenz et al. 2023). We have now added this explanation and references also to the revised manuscript:

Line 94: “We have previously published the development and full physicochemical characterisation of PCL/PEO and PCL/PEO/CAM ES fibers,^18^ and investigated the antimicrobial effect of the antibiotic-loaded fibers against various wound pathogens and using different antimicrobial assays^18,21,22^. In the present study, we tested these ES materials in two different cell lines using indirect extract exposure and direct contact cytotoxicity evaluations.”

References:

Preem, L. *et al.* Interactions between Chloramphenicol, Carrier Polymers, and Bacteria-Implications for Designing Electrospun Drug Delivery Systems Countering Wound Infection. *Molecular Pharmaceutics* **14**, 4417–4430 (2017). DOI: [10.1021/acs.molpharmaceut.7b00524](https://doi.org/10.1021/acs.molpharmaceut.7b00524)

Preem *et al.* Monitoring of Antimicrobial Drug Chloramphenicol Release from Electrospun Nano- and Microfiber Mats using UV Imaging and Bacterial Bioreporters. *Pharmaceutics* **11**, 487 (2019). DOI: [10.3390/pharmaceutics11090487](https://doi.org/10.3390/pharmaceutics11090487)

Lorenz, K. *et al.* Development of In Vitro and Ex Vivo Biofilm Models for the Assessment of Antibacterial Fibrous Electrospun Wound Dressings. *Mol. Pharmaceutics* **20**, 1230–1246 (2023). DOI: [10.1021/acs.molpharmaceut.2c00902](https://doi.org/10.1021/acs.molpharmaceut.2c00902)

**Introduction:**

**Q1: Line 36, “various novel biomaterials” include some examples and potential applications.**

R1: We have added some examples and potential applications as suggested by the Reviewer, the revised manuscript now reads:

Line 36: “Some of the examples of biomaterials are implants, heart valves, stents, lenses, prostheses, wound dressings etc^1^. Biomaterials are widely used in regenerative medicine, tissue engineering, orthodontics and drug delivery^2^.”

References:

Ratner, B. D. Biomaterials: Been There, Done That, and Evolving into the Future. *Annu. Rev. Biomed. Eng.* **21**, 171–191 (2019). DOI: [10.1146/annurev-bioeng-062117-120940](https://doi.org/10.1146/annurev-bioeng-062117-120940)

Ajmal, S., Athar Hashmi, F. & Imran, I. Recent progress in development and applications of biomaterials. *Materials Today: Proceedings* **62**, 385–391 (2022). DOI: [10.1016/j.matpr.2022.04.233](https://doi.org/10.1016/j.matpr.2022.04.233)

**Q2: Line 38, What does “controversial reports can be found” mean?**

R2: We have modified the text and removed this statement, and the text now reads as follows:

Line 38: The terms and definitions of “biomaterials” have changed a lot over the past decades, and nowadays artificial organs, tissue scaffolds and delivery vehicles for cells, genes and active pharmaceutical ingredients can all be classified as biomaterials^3^.

Reference:

Masaeli, R., Zandsalimi, K. & Tayebi, L. Biomaterials Evaluation: Conceptual Refinements and Practical Reforms. *Ther Innov Regul Sci* **53**, 120–127 (2019). DOI: [10.1177/2168479018774320](https://doi.org/10.1177/2168479018774320)

**Q3: Line 42, “The major mutual characteristic to be termed as a …………. Should be prepared for a specific purpose 1-4”, the statement is complex. Please simplify the statement.**

R3: We have simplified the definition in order to make it clearer, the statement now reads as follows:

Line 42: “Biomaterial is a material/substance that has been engineered to become in contact with living tissues for therapeutic or diagnostic purposes.”

**Q4: Line 47, “a high surface area for efficient drug release”, high surface area provide high drug or molecule loading capacity not the release.**

R4: Thank you for this remark, by accident we only had “surface area” in the text making our statement invalid. The high surface area to volume ratio is one of the key parameters that may largely affect the drug release properties. That is the reason why “nano” systems provide advantages over the larger particles (or fibers with larger diameters) and these nanotechnological biomaterials enable to increase the dissolution rate and release of poorly water-soluble drugs from ES fibers. We have revised the manuscript as follows:

Line 47: “ES mats have several prominent features, such as a porous structure, the abilities to incorporate drugs, including antibiotics, into fiber structures, and a high surface area to volume ratio for efficient drug release^8^.”

Reference:

Luraghi, A., Peri, F. & Moroni, L. Electrospinning for drug delivery applications: A review. *Journal of Controlled Release* **334**, 463–484 (2021). DOI: 1[0.1016/j.jconrel.2021.03.033](https://doi.org/10.1016/j.jconrel.2021.03.033)

**Q5. Line 52, “Providing enough oxygen and moisture to …” this does provide optimal conditions for bacterial growth as well.**

R5: Indeed, these are also very good conditions for bacteria to grow, but it is well known that bacteria and eukaryotic cells prefer to live in somewhat similar environments. Properties such as good moisture retention, air permeability and a fibrous surface are precisely what is sought in wound dressings. Unfortunately, there is no way to avoid the fact that in addition to supporting the wound healing, wound dressings also have bacterial growth-promoting properties. For this reason, we are developing wound dressing materials in which we can incorporate antibiotics and other antimicrobial agents that are safe for eukaryotic cells and support the wound healing but inhibit the bacterial growth.

We added the following sentence to the revised manuscript:

Line 55: “More importantly, the presence of antimicrobial agent in ES wound mats is relevant as this inhibits the growth of bacteria which is also more favoured in these mentioned environmental conditions developed.”

**Q6: Line 69, “with test material” not tested material.**

R6: We replaced the word “tested” with “test” (line 75).

**Q7: Line 75-78, “after an exhaustive literature …….. (MTT, MTS) has been used.” That is true. But MTT/MTS are used for cell metabolic activity assessment and when standardized with cell count supports cell viability/toxicity assessment as well. But in general, MTT/MTS assays are used in conjunction with fluorescence or other biological assessment procedures.**

R7: We agree that it is beneficial to use MTT/MTS assays together with other suitable methods to gather more relevant information. Therefore, we used RTCA and microscopy with fluorescent staining, in addition to the MTS assay. However, the ISO 10993-5 standard, which is the basis of safety testing for novel materials, recommends choosing and using only one method; MTT is one such method in the ISO standard list. We agree completely with the reviewer that different methods have to be combined and this was one of the reasons for preparing the current manuscript.

**Results:**
**Q1. Line 99-104, “SEM images and size analysis revealed ……….” 0.94 ± 1.56 micron for pristine and 1.03 ± 1.30 micron for CAM loaded ES fiber. That is a big standard deviation as big or bigger than measured average diameter suggesting non homogenous diameter as reported by authors. Based on this statement, how the authors can rely on their biological analysis. This large variation in ES fiber diameter questions all analyses and investigations.**

R1: We would like to explain this more in order to illustrate and provide evidence that these analyses and investigations are valid. These model ES fiber mats were prepared newly and specifically for these biological analyses. We used SEM analysis and characterised the ES mats immediately after electrospinning and confirmed the reproducibility of the ES process based on the analysis of morphology and fiber diameter (fiber diameter size distribution). As can be seen in Figure 1a and b, the mats consisted of fibers with very small fiber diameters and large fiber diameters, and as can be seen from histograms bimodal size distribution was seen. Most of the smaller fibers had the fiber diameter of approximately 300 nm and most of the larger fibers were above 2 micrometers. Hence the average fiber diameter (approximately 1 µm) does not illustrate that the mats consisted of fibers where most of the fibers had the fiber diameter of 1 µm and with huge error. The actual fiber size distribution is much more illustrated by the histograms. Such fibrous mats actually resemble a lot our own skin extracellular structure and can be considered as desired structure for the cells (specifically when the mats are used as skin scaffolds).

In order to guide the reader and describe the fiber mats morphology and fiber diameters we revised the text as follows:

Line 110: “SEM images and size analysis revealed a wide variation in the fiber diameters throughout the mats (**Figs. 1 a** and **b**). The diameter of the fibers was in the range between 0.1 µm and 8 µm, where most of the fibers where in the range between 0.1 µm and 0.5 µm for both PCL/PEO and PLC/PEO/CAM fiber mats. The histograms of fiber size distribution characterise the fibers obtained better as compared to the arithmetic mean diameter of the fibers. The structure of the obtained ES fiber mats resembles the skin´s extracellular matrix structure with heterogeneous fiber sizes.”

**Q2. This variation in diameter can significantly alter the physical and mechanical properties of ES fibers, not reported in manuscript.**

R2: We agree that the variance in the fiber diameter can change the physical and mechanical properties of the ES fiber material. We have previously conducted and reported the full physicochemical characterisation of the used ES fiber materials (Preem et al. 2017 and 2019). As the materials and formulations were not modified, we indeed did not test all these properties in depth for the present manuscript, in this case SEM and drug content analyses were tested for each ES mat used for biological experiments. We decided to focus on *in vitro* safety testing assays.

References:

Preem, L. *et al.* Interactions between Chloramphenicol, Carrier Polymers, and Bacteria-Implications for Designing Electrospun Drug Delivery Systems Countering Wound Infection. *Molecular Pharmaceutics* **14**, 4417–4430 (2017). DOI: [10.1021/acs.molpharmaceut.7b00524](https://doi.org/10.1021/acs.molpharmaceut.7b00524)

Preem *et al.* Monitoring of Antimicrobial Drug Chloramphenicol Release from Electrospun Nano- and Microfiber Mats using UV Imaging and Bacterial Bioreporters. *Pharmaceutics* **11**, 487 (2019). DOI: [10.3390/pharmaceutics11090487](https://doi.org/10.3390/pharmaceutics11090487)

**Q3. Line 106, raw material’s batch variability has been reported and known but the trend can be investigated or speculated with couple of batches.**

R3: Thank you, this is of interest to us as well and will be investigated in our further studies. We actually have more data (and hypotheses), but this is out of the scope of the present manuscript.

**Q4: Line 162, as both the cell types have different size and morphology, the confluency in the well or on well surface will be different with time even after same seeding cell density because cell might have different proliferation rate.**

R4: Exactly, we have discussed this also in our manuscript in order for the readers to consider this while they are designing their *in vitro* safety assays.

**Q5: Line 187, if the authors believed the different passage number can affect the behavior which is well known for primary cells. The authors should have repeated with study with same P number.**

R5: We conducted all the experiments with primary cells using the suggested passage numbers by the authorised agencies in cell culture and production (ATCC, UK Health Security Agency). A passage number refers to the number of times the cells in the culture have been transferred from one vessel into another (subcultured). For primary cells indeed it has been suggested to use up to 8-12 passages, after which the cell growth and behaviour may start to change. While analysing the data we saw some differences in the results. Our hypothesis was that as all other conditions were kept constant then this might be due to the passage number differences. We have now repeated the RTCA experiments as suggested by the Reviewer and used “more similar” passage number as it was not possible to use exactly the same passage number for the cells available. These obtained results are now presented on the **Figs 3** panel **d**. Also, our hypothesis was confirmed. The overall shape and tendency of the graph remained the same for cells with different passage number, but the cell index number was different (See comparison **Fig S1** below). We put the results obtained with cells with higher passage number to the Supplementary Material (**Fig S1**) to highlight this effect since it is not much discussed in the articles where the safety of ES fibers is tested. We added an explanatory paragraph in the revised manuscript, and the text reads now as follows:

Line 201: “One aspect that needs to be considered in such an experimental setup is the nature and specificity of the cell line used. There was a clear difference in the cell index values when experiments were conducted with PF cells using two different passage numbers (**Figs. 3d** and **S1**). The experiments were performed with PF cells with passage number 5 (**Fig. 3d**) and 12 (**Fig. S1**). The cell index values were higher when using the cells with lower passage number (**Fig. 3d**) and phenomenon was reversed when using the cells with higher passage number (**Fig. S1**), but the overall shape of the graph remained the same. The effect of passage number on the experimental results for primary cells has been described previously in the literature^29,30^. The effect of passage number on BHK-21, an immortalised cell line, was not observed in the experiments.”

**Fig S1.** Results of RTCA indirect ES fiber mat extract exposure assay performed on PF cells at passage numbers 12 (a) and 5 (b). The overall graph shape was similar, but the cell index value was lower when using cells with higher passage numbers.

Reference:

<https://www.atcc.org/resources/technical-documents/reference-strains-how-many-passages-are-too-many> (last view: 25.04.2024)

<https://www.culturecollections.org.uk/training-and-support/cell-line-passage-numbers-explained/> (last view: 25.04.2024)

**Q6. Line 226, metabolic activity can be a measure of comparable number cells but more metabolically active or more cells with lesser metabolic activity based on if cells like the surface or not.**

R6: Correct, MTS activity may be related with the number of cells as well as their metabolic activity. In some cases, the cell number may be constant, but cells are more metabolically active and other way around as well (more cells, but metabolic activity is not changed). That is also the reason why MTS analysis is good to be used in parallel with the microscopic analysis of the samples. This allows making conclusive statements about cell behaviour. We modified our statement, and the text reads now as follows:

Line: 243: “This could indicate that the old growth medium (medium where cells have been grown for 72 h) limits MTS activity, because cells might not have enough nutrients to grow and therefore their metabolic activity is decreased.”

We also added this explanation into the Discussion part:

Line: 425: “For this reason, it is necessary to consider cell seeding density, because it influences outcome of tetrazolium salt assays^46^. Moreover, metabolic activity of the cells is influenced by the excess or deficiency of nutrients^56^ and larger number of cells with lesser nutrients results in decreased metabolic activity which affects the MTS assay results.”

**Q7: Line 242, ES fibers are known to be porous which can adsorb MTS reagent leading to false signal.**

R7: Yes, indeed, ES fibers can also adsorb the MTS reagent or other colorimetric assay dyes. This is one aspect to keep in mind when designing the *in vitro* cell studies to test the ES material safety.

In order to rule out the possibility of MTS reagent adsorption and/or absorption to the tested ES fibers we performed a simple experiment, described in Supplementary materials. In short, the BHK-21 cells were seeded on a 24-wellplate and incubated for 24 h. Then, the ES fibers and MTS reagent were placed into the wells and incubated for 1 h. MTS OD was then measured. After that, the leftover cell culture medium and the ES fibers were transferred to a new plate to remove the cells and incubated for another hour. After each step the ES fibers were also photographed to capture the colour change caused by the MTS reagent binding. We visually saw only small change in the ES fiber colour and the MTS assay results showed no difference between groups where MTS reagent was incubated together with cells and ES fibers compared to the control group where MTS reagent was incubated only with cells. These two tests showed that no drastic MTS reagent binding occurred (**Figures S2** and **S3**).

**Fig S2.** Results of possible MTS reagent binding to the ES fibers in the presence of BHK-21 cells. No significant differences were observed between the groups.

**Fig S3**. Photographs of the tested ES fibers taken after each experimental step to visually detect the possible adsorption and/or absorption of the MTS reagent. No drastic colour change was observed, indicating that the MTS reagent may only slightly adsorb and/or absorb the tested ES fibers.

We added the following explanations into the manuscript and additional information to the **Supplementary materials**:

Line 261: Since the ES fiber materials are also known to be porous it is important to keep in mind that some level of MTS reagent adsorption and/or absorption may occur. We tested our ES fibers to rule out these possibilities. The results showed that no drastic MTS reagent binding occurred (**Fig. S2**). Visually only small colour changes of the tested ES fibers occurred (**Fig. S3**). The experiment and its results are described in the Supplementary material.

In the Discussion part we have already previously added a paragraph about the material reagent binding properties and why we decided to alter the result analysis and interpretation:

*One additional aspect to consider when setting up cytotoxicity testing experiments is the fact that the tested material itself can bind some amount of the testing substrate compound or product, leading the experimental results to be biased toward the cytotoxicity side^12^. This is one reason why using the MTS activity fold increase gives us a better understanding of viability and toxicity compared to the percentage calculation or comparing the raw MTS activities.*

We added an explaining sentence:

Line 419: “Since ES fiber materials are also known to be porous, reagent binding may occur when using colorimetric assays and it could lead to incorrect interpretation of the results.”

**Q8: Line 248, with different cells size and non-uniform ES fiber diameters, the cell distribution over the ES Fiber mat will be irregular making the any type of biological assessment a big challenge.**

R8: This is why we decided to write this article, to show that testing novel biomaterials is not as easy task despite the ISO standards and guidelines. It is not often discussed in the available literature. We agree that any kind of biological testing is a huge challenge, and when adding different cell lines to the mix, the picture becomes very complex. Our manuscript is written in order to give guidance to the researchers to what steps to consider and what tests could be used, and what are the “errors” that can be avoided when careful design of experiments has been developed.

**Q9: Line 253, “a fold increase in MTS activity” this is not clear.**

R9: It was defined in the next sentences what is “a fold increase in MTS activity”:

*The fold increase in MTS activity was calculated from the 24 h and 48 h time point OD results by dividing the 48 h MTS activity by the 24 h activity. The obtained value provides a better understanding of cell growth over time*.

We modified the sentences and added additional explanations, the text now reads as follows:

Line 276: “The fold increase in MTS activity was calculated from the 24 h and 48 h time point MTS assay OD 490 nm results using the following formula:

$$Fold increase in MTS activity=\frac{48 h MTS OD}{24 h MTS OD}$$

The obtained value provides a better understanding of cell growth over time as it describes the MTS activity increase over time. The authors used this in context as a number of estimation of cell divisions that took place between the measurement time-points.”

**Q10: Line 285, the ES fiber mat may not be cytotoxic but the biocompatibility or likeability for fiber mat for both cell types might be different, leading to modulating cell attachment, cell morphology, and or cell metabolic activity. Also, as reported by authors the ES fiber diameter was not homogenous which can tailor the ES fiber or fiber mat interaction with cells.**

R10: Yes, that was something we also discussed in the Discussion section.

*Cell attachment is a dynamic process which changes over time during the course of an experiment, and cell morphology is affected by surface smoothness, stiffness, and chemical composition^39,49^. Not only do cells change their properties, but the ES fiber mat also changes its properties in an aqueous environment. We have shown that cells change their morphology when grown on top of ES fiber mats compared to the glass used as a control (****Figs. 6*** *and* ***7)****. The size and shape of cells differ, but growth on glass is not a physiological condition for the cells, and higher activation of actin filaments was observed for the cells grown on a glass surface compared to cells grown on the fiber mat surface, presumably because of the greater need for attachment on a glass surface. Compared to plastic or glass, ES fiber material is a softer material and therefore affects the cell morphology. Soft surfaces have been shown to reduce the activity of stress actin filaments in cells^40,50–52^.*

It has been previously reported by other authors that cell attachment and morphology changes with the structure and stiffness of the material on which cells are growing. We also understand that the heterogeneity in fiber size can change the interaction and behaviour of the cells, but we do not see it as a disadvantage but as an advantage, when for example the fibers are used as skin scaffolds. ES fibers are often compared with the extracellular matrix, which also does not have homogenous structure and cells in the scar tissue have different cell morphology compared to the normal healthy cells.

We added some additional explanation into the Discussion part:

Line 409: “Moreover, ES fibers have a structure resembling skin´s own extracellular matrix structure and the bioactivity of the cells changes when grown on ES fiber material^53,54^.”

References:

Yeung, T. *et al.* Effects of substrate stiffness on cell morphology, cytoskeletal structure, and adhesion. *Cell Motil. Cytoskeleton* **60**, 24–34 (2005). DOI: [10.1002/cm.20041](https://doi.org/10.1002/cm.20041)

Hopp, I. *et al.* The influence of substrate stiffness gradients on primary human dermal fibroblasts. *Biomaterials* **34**, 5070–5077 (2013). DOI: [10.1016/j.biomaterials.2013.03.075](https://doi.org/10.1016/j.biomaterials.2013.03.075)

Solon, J., Levental, I., Sengupta, K., Georges, P. C. & Janmey, P. A. Fibroblast Adaptation and Stiffness Matching to Soft Elastic Substrates. *Biophysical Journal* **93**, 4453–4461 (2007). DOI: [10.1529/biophysj.106.101386](https://doi.org/10.1529/biophysj.106.101386)

Missirlis, D., Haraszti, T., Heckmann, L. & Spatz, J. P. Substrate Resistance to Traction Forces Controls Fibroblast Polarization. *Biophysical Journal* **119**, 2558–2572 (2020). DOI: [10.1016/j.bpj.2020.10.043](https://doi.org/10.1016/j.bpj.2020.10.043)

Mungenast, L. *et al.* Electrospun decellularized extracellular matrix scaffolds promote the regeneration of injured neurons. *Biomaterials and Biosystems* **11**, 100081 (2023). DOI: [10.1016/j.bbiosy.2023.100081](https://doi.org/10.1016/j.bbiosy.2023.100081)

Slivac, I. *et al.* Bioactivity Comparison of Electrospun PCL Mats and Liver Extracellular Matrix as Scaffolds for HepG2 Cells. *Polymers* **13**, 279 (2021). DOI: [10.3390/polym13020279](https://doi.org/10.3390/polym13020279)

**Q11: Line 300, what are different physical, mechanical, and chemical characterization techniques. Authors should have included the physical, mechanical, and chemical assessment for ES fiber pristine and CAM loaded.**

R11: The purpose of this article was purely to test the *in vitro* safety of the ES fiber materials and how different aspects of it affect the results. Previously developed materials were used for this purpose. The full physicochemical and antibacterial characterisation of these ES fiber materials has been reported in previously published articles by our research group.

References:

Preem, L. *et al.* Interactions between Chloramphenicol, Carrier Polymers, and Bacteria-Implications for Designing Electrospun Drug Delivery Systems Countering Wound Infection. *Molecular Pharmaceutics* **14**, 4417–4430 (2017). DOI: [10.1021/acs.molpharmaceut.7b00524](https://doi.org/10.1021/acs.molpharmaceut.7b00524)

Preem *et al.* Monitoring of Antimicrobial Drug Chloramphenicol Release from Electrospun Nano- and Microfiber Mats using UV Imaging and Bacterial Bioreporters. *Pharmaceutics* **11**, 487 (2019). DOI: [10.3390/pharmaceutics11090487](https://doi.org/10.3390/pharmaceutics11090487)

To clarify that this article focuses only on *in vitro* safety studies of ES fiber materials, we have added this sentence to the Introduction section. The text reads now as follows:

Line 94: “We have previously published the development and full physicochemical characterisation of PCL/PEO and PCL/PEO/CAM ES fibers,^18^ and investigated the antimicrobial effect of the antibiotic-loaded fibers against various wound pathogens and using different antimicrobial assays^18,21,22^. In the present study, we tested these ES materials in two different cell lines using indirect extract exposure and direct contact cytotoxicity evaluations.”

**Q12: Line 302, the biological investigation is not limited to toxicity/cytotoxicity and inflammation assay. Assays such as ROS regeneration, DNA damage, DNA/cell number quantification, proliferation assay, attachment assay, different pathway studies are equally important.**

R12: We agree. Therefore, we added these to the list too. The sentence reads now as follows:

Line 327: “However, biological methods to test toxicity are extremely important properties, but we cannot also forget proliferation and attachment assays, inflammatory response, ROS regeneration and DNA damage assays.”

**Q13: How about the degradation rate or degradability of ES PEO/PCL fiber with or without CAM.**

R13: From previous studies we know that CAM is released quickly from PCL/PEO/CAM ES fibers and PEO dissolves in aqueous solutions, but PCL is very durable and remains the same for longer time-periods. We have added explanations about this to the manuscript and linked it with the choice of the indirect extract exposure method. The text reads now as follows:

Line 365: “From our previous studies, we know that CAM is released fast from the PCL/PEO/CAM ES fibers and much of the PEO is dissolved, but PCL remains the same in aqueous solution^18,21^. Therefore, it is important to use the extract exposure method to understand the toxic effects of the released substances on eukaryotic cells. We showed that the substances released from the PCL/PEO and PCL/PEO/CAM ES fiber mats during the specified time period were safe for the cells (**Figs. 2** and **3**).”

References:

Preem, L. *et al.* Interactions between Chloramphenicol, Carrier Polymers, and Bacteria-Implications for Designing Electrospun Drug Delivery Systems Countering Wound Infection. *Molecular Pharmaceutics* **14**, 4417–4430 (2017). DOI: [10.1021/acs.molpharmaceut.7b00524](https://doi.org/10.1021/acs.molpharmaceut.7b00524)

Preem *et al.* Monitoring of Antimicrobial Drug Chloramphenicol Release from Electrospun Nano- and Microfiber Mats using UV Imaging and Bacterial Bioreporters. *Pharmaceutics* **11**, 487 (2019). DOI: [10.3390/pharmaceutics11090487](https://doi.org/10.3390/pharmaceutics11090487)

**Conclusion:
The ES fiber under investigation are suggested to be antimicrobial so, the antimicrobial property should be assessed with at least some of the bacterial species causing wound infection such as *Pseudomonas aeruginosa, Staphylococcus aureus, Klebsiella pneumoniae, Enterococcus faecalis,* and *Acinetobacter baumannii*.**

The aim of the article was to develop methods for testing the safety of ES materials *in vitro* and draw attention to aspects and interpretation of the results that may need to be altered when using already available frequently used methods.

The ES fibers used in this study have previously been tested for their antimicrobial activity against these bacteria:

*E. coli* non-pathogenic strain - Preem, L. *et al.* Interactions between Chloramphenicol, Carrier Polymers, and Bacteria-Implications for Designing Electrospun Drug Delivery Systems Countering Wound Infection. *Molecular Pharmaceutics* **14**, 4417–4430 (2017). DOI: [10.1021/acs.molpharmaceut.7b00524](https://doi.org/10.1021/acs.molpharmaceut.7b00524)

*S. aureus* pathogenic strain – Preem *et al.* Monitoring of Antimicrobial Drug Chloramphenicol Release from Electrospun Nano- and Microfiber Mats using UV Imaging and Bacterial Bioreporters. *Pharmaceutics* **11**, 487 (2019). DOI: [10.3390/pharmaceutics11090487](https://doi.org/10.3390/pharmaceutics11090487)

*E. coli,* *S. aureus* and *S. epidermidis* wound related pathogenic strains – Lorenz, K. *et al.* Development of In Vitro and Ex Vivo Biofilm Models for the Assessment of Antibacterial Fibrous Electrospun Wound Dressings. *Mol. Pharmaceutics* **20**, 1230–1246 (2023). DOI: [10.1021/acs.molpharmaceut.2c00902](https://doi.org/10.1021/acs.molpharmaceut.2c00902)

Additionally, we have two separate publications in writing which will be published in the near future where the PCL/PEO and PCL/PEO/CAM ES fiber materials are tested in biorelevant *ex vivo* infected wound model and on *in vitro* polymicrobial infection model.
